# Supplementary material for: Overlooked but Serious Gallbladder Disease during Extracorporeal Membrane Oxygenation: A Retrospective Analysis
Source: J Clin Med. 2022 Apr 14;11(8):2199. doi: 10.3390/jcm11082199 (PMC9031104; doi:10.3390/jcm11082199)
Supplement: Supplementary file 1 [file jcm-11-02199-s001.zip › jcm-1627169-supplementary.pdf]

Table S1. Other extracorporeal membrane oxygenation complications related to gallbladder disease in the propensity-matched cohort

**Variables**	**G group (*n* = 62)**	**N group (*n* = 124)**	***p***
Oxygenator thrombosis	12 (19.4)	23 (18.5)	0.894
PTE	4 (6.5)	4 (3.2)	0.307
DVT	1 (1.6)	1 (0.8)	0.615
Hyperbilirubinemia	39 (62.9)	54 (43.5)	0.013
Cannula site bleeding	11 (17.7)	14 (11.3)	0.224
DIC	3 (4.8)	2 (1.6)	0.200
GI hemorrhage	5 (8.1)	10 (8.1)	1.000
Severe Hemolysis (plasma Hb > 100 mg/ dL)	19 (30.6)	3 (2.4)	<0.001
Retroperitoneal bleeding	3 (4.8)	0	0.014
Infection	35 (56.5)	73 (58.9)	0.753

DIC, disseminated intravascular coagulation; DVT, deep vein thrombosis; GB, gallbladder; G group, a group with GB disease; GI, gastrointestinal; Hb, hemoglobin; N group, a group without GB disease; PTE, pulmonary thromboembolism.

Data was presented as *n* (%).
